# Proton pump inhibitors for the treatment of cancer in companion animals

**DOI:** 10.1186/s13046-015-0204-z

**Published:** 2015-09-04

**Authors:** Megan Walsh, Stefano Fais, Enrico Pierluigi Spugnini, Salvador Harguindey, Tareq Abu Izneid, Licia Scacco, Paula Williams, Cinzia Allegrucci, Cyril Rauch, Ziad Omran

**Affiliations:** School of Veterinary Medicine and Science, University of Nottingham, College Road, Sutton Bonington, LE12 5RD UK; Department of Therapeutic Research and Medicines Evaluation, National Institute of Health, Viale Regina Elena 299, 00161 Rome, Italy; SAFU, Regina Elena Cancer Institute, Via delle Messi d’ Oro 156, Rome, Italy; Institute for Clinical Biology and Metabolism, c) Postas 13, 01004 Vitoria, Spain; College of Pharmacy, Umm Al-Qura University, Al-Abidiyya, 21955 Makkah, Kingdom of Saudi Arabia; Equivet Roma Hospital, Equine Veterinary Clinic, Via di Torre di Sant’Anastasia 83, 00134 Rome, Italy

**Keywords:** pH, Cancer, Proton pump inhibitors, Chemoresistance, Metastasis

## Abstract

The treatment of cancer presents a clinical challenge both in human and veterinary medicine. Chemotherapy protocols require the use of toxic drugs that are not always specific, do not selectively target cancerous cells thus resulting in many side effects. A recent therapeutic approach takes advantage of the altered acidity of the tumour microenvironment by using proton pump inhibitors (PPIs) to block the hydrogen transport out of the cell. The alteration of the extracellular pH kills tumour cells, reverses drug resistance, and reduces cancer metastasis. Human clinical trials have prompted to consider this as a viable and safe option for the treatment of cancer in companion animals. Preliminary animal studies suggest that the same positive outcome could be achievable. The purpose of this review is to support investigations into the use of PPIs for cancer treatment cancer in companion animals by considering the evidence available in both human and veterinary medicine.

## Background

In recent years the role of pH in cancer has received increasing attention from researchers worldwide. It is becoming clear that pH plays an important role in the survival mechanisms of mammalian cancer cells and that it is implicated in the resistance to drugs that some cancer types develop during chemotherapy [1]. Over the last 40 years the number of human cancer deaths and new cases per year has generally remained static, but the survival times have been increasing gradually mainly due to improved screening methods, and earlier detection rather than advancements in treatment [2]. Currently, the main reason for treatment failure is the development of drug resistance [3], thus forcing scientists and clinicians to rethink the way cancer is treated. Historically, clinicians have used chemotherapy protocols to eradicate the growth of fast proliferating tumour cells. However, the dose levels required to overcome the developing resistance cause human patients discomfort which can reach unacceptable levels in the more advance stages of the disease, ultimately resulting in the treatment failing to keep the cancer under control. It is well known that cancer is not only a genetic disease, but a condition which results from clonal selection of metabolic changes conferring cancer cells a growth advantage [4]. In this context, it has been demonstrated that extracellular pH plays an important role in drug resistance and malignant progression [1]. Targeting tumour pH can be therefore considered a valid and novel therapeutic strategy [5].

### pH in cancer

Conventional therapies aimed at targeting proliferating cancer cells often do not take in account cancer complexity and tumour heterogeneity. In recent years, scientists have studied cancer at the molecular level and investigated the phenotypic changes and markers in different types of cancer [6]. One of the most important phenotypic changes is the cancer cell’s ability to change the extracellular acidity due to an altered glycolysis pathway [7]. Nearly a century ago, Otto Warburg recognised the acidification of the tumour microenvironment [8], postulating that cancer cells use the less efficient anaerobic glycolysis pathway even in the presence of oxygen, now known as the Warburg hypothesis. This alternative pathway results in an intracellular accumulation of lactic acid which would lead to cellular death if not removed. As such, the cancer cells develop survival mechanisms to cope with this decreased intracellular pH which allow them to survive in an adapted tumour microenvironment [1].

**Fig. 1:**
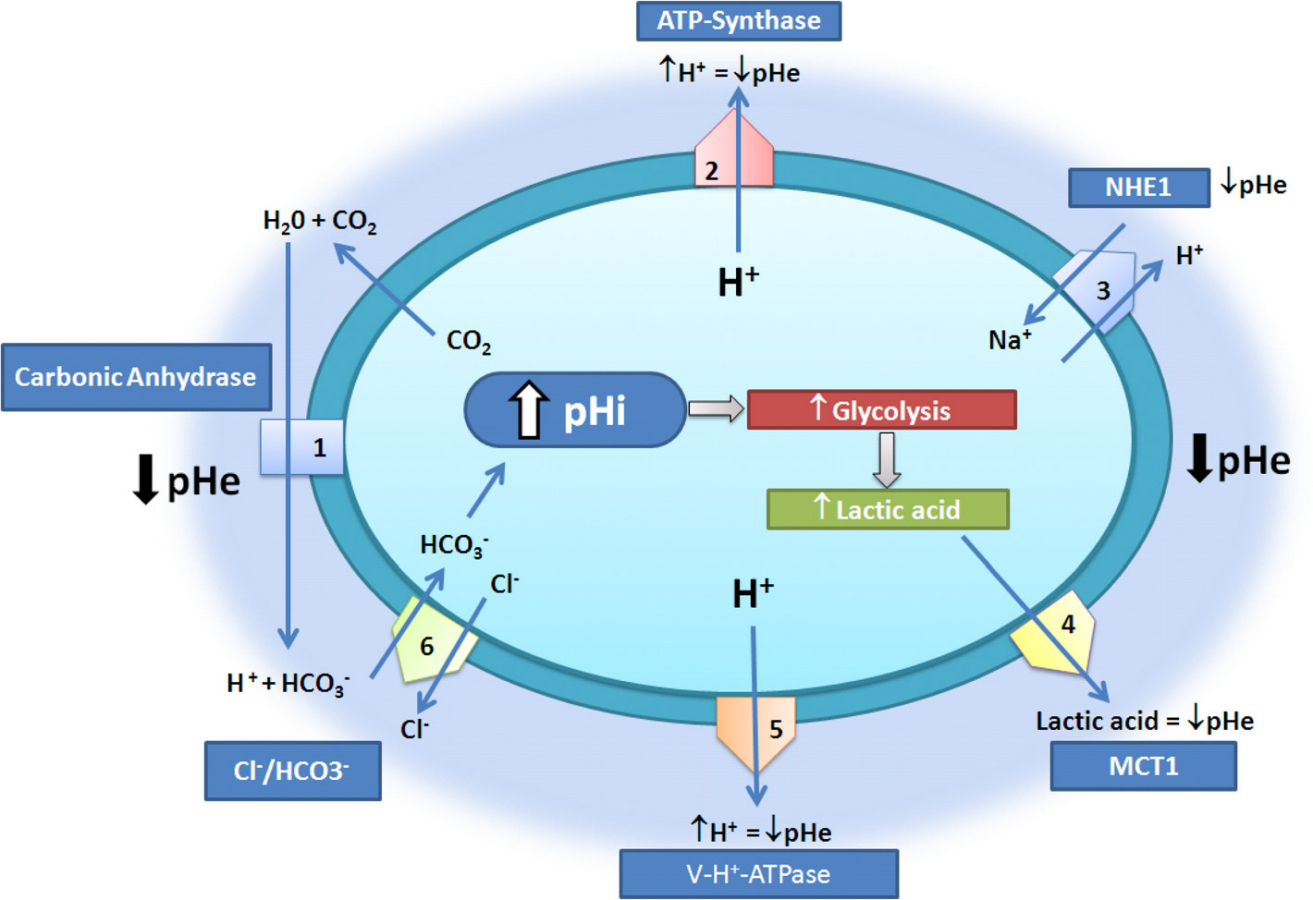
pH regulation in cancer cells. A number of proteins and chemical reactions regulates pH in cells. The present review focuses on NHE1 and V-ATPase. 1: CAs, 2: ATP-Synthase, 3: NHE1, 4: MCTs, 5: V-H^+^-ATPase, 6: Cl^−^/HCO_3_^−^. pHi = Intracellular pH. pHe = Extracellular pH [28]

One way cancer cells avoid the accumulation of intracellular acid is by the up-regulation of proton pumps (PP) and transporters (PTI) responsible for the removal of hydrogen ions from the intracellular compartments or cytosol to the extracellular microenvironment. Several biological mechanisms exist to export hydrogen ions out of the cells and to affect the extracellular pH including the carbonic anhydrase (CA) enzymes (and in particular CA IX) [9], the monocarboxylic transporters (MCT), the Vacuolar-H + −ATPase (V-ATPase) and the Na+/H+ transporters (NHE) [1] (Fig. 1).

Of all the 16 isoforms of CA, CA IX seems to be prevalent in cancer. Whilst in non-disease states CA IX expression is limited to the gut epithelium (namely the basolateral surfaces of the cryptic enterocytes), CA IX is ectopically expressed in a variety of neoplastic tissues and knockdown of CA IX expression (or its chemical inhibition) has been shown to reduce primary tumor growth, and decrease drug resistance. CA catalyzes the removal of a water molecule from carbonic acid group and as a result can affect the pH of the extracellular environment [9] (Fig. 1).

Lactate is a byproduct from glucose breakdown through glycolysis in anaerobic conditions. As a means of regenerating oxidized NAD^+^, lactate dehydrogenase catalyzes the conversion of pyruvate to lactate in the cytosol. The transport of the acid lactate is ensured via specific transporters known as monocarboxilate transporters. Of all the 14 MCTs, expression and regulation of MCT1, MCT2, MCT3 and MCT4 have been reported in a wide variety of cancers. Upon transport, the proton and lactate anion are associated as they cross the cell membrane resulting in a net efflux of proton outside cancer cells [10] (Fig. 1).

V-ATPases are proton (H^+^) pumps that actively transport H^+^ out of the cell using ATP [9, 11]. They are membrane proteins expressed at the level of the plasma membrane and intracellular membrane organelles. V-ATPases are composed of V_0_, a membrane complex, and V_1_, a soluble domain [12]. V_0_ is the integral site responsible for the movement of H^+^ across the plasma membrane, whereas V_1_ is responsible for ATP hydrolysis [13] (Fig. 1).

Na^+^/H^+^ antiporters remove H^+^ in exchange for Na^+^(1H^+^:1Na^+^). NHE is composed of two complexes, the C-terminal and the N-terminal regulating the activity of the cytoskeleton and the ion transportation, respectively [14]. Different isoforms exist, with NHE1 being ubiquitous and NHE2 and NHE3 largely expressed in the kidney and intestine [12] (Fig. 1).

The ability to efflux protons from cells and therefore change the extracellular pH provides an advantage to cancer cells. For instance, the weakly basic cytotoxic drugs (e.g. doxorubicin) used in chemotherapy protocols are neutralised by the lowered pH. In this way, drugs are protonated when they reach the acidic barrier surrounding the cell and are subsequently unable to pass through the cell membrane [15]. V-ATPases have been shown to be associated with multidrug resistance which can be reversed by using inhibitors of these proton pumps [5].

Other survival mechanisms that cancer cells have developed as a result of the acidic microenvironment are the up-regulation of drug transporters at the cell membrane [16], and alteration of the biophysical properties of the membrane [17–19] which are responsible for the efflux of the cytotoxic drugs and the onset of drug resistance. In order to overcome these challenges, therapies use high concentrations of drugs from the start of the therapy or use another additional drug, which inevitably result in more negative side effects for patients. Moreover, the tumour acidic environment negatively affects the immune system, leading to a state of local anergy that prevents the immune cells from exploiting the tumour shrinkage and the exposure of tumour antigens that follows a successful chemotherapy [20].

The acidic tumour microenvironment has also been associated with the degree of cancer aggressiveness. It has been reported that the lower the pH surrounding the cell, the more likely is the chance of malignancies with higher degree of invasiveness [16]. This can be due to the increased lysosomal exocytosis of intracellular proteases which occurs secondary to the acidic microenvironment. These proteases can affect the extracellular matrix in the surrounding tissue and may induce the invasion of the surrounding tissues [16, 21]. Some studies suggest that cancers with low metastatic potential utilise mostly Na+/H+ exchangers, whereas highly metastatic cancers use V-ATPases [22]. Therefore, the ability to manipulate the acidity of the cancer cells microenvironment presents novel therapeutic opportunities to prevent the progression and spread of cancer [15, 23–25].

### Proton pump inhibitors

In an attempt to manipulate and neutralise this acidic microenvironment, proton pump inhibitors (PPIs) have been used to target the V-ATPase pumps present on the cell membrane. The treatment leads to an alkalisation which subsequently reduces the degree of protonation of chemotherapy drugs rendering them far more effective at much lower doses [26].

The specific inhibitor of V-ATPase pumps Bafilomycin has been evaluated for its antineoplastic activity [27] but it was demonstrated to be highly toxic to normal cells even at low doses [28]. Other studies have also looked at the use of the diuretic Amiloride to block the transport of protons out of the cell via the NHE transporter [16], and several studies have demonstrated anti-tumours and anti-metastatic activity in cancer cells and animal models [29].

PPIs used as irreversible blockers of the gastric H+/K+ ATPase pump have been also demonstrated to be effective on V-ATPases at higher concentrations [30, 31]. Because of their similarity, these inhibitors which are routinely used as antacids, are at the most advanced stages of clinical use compared to other H+ pump inhibitors [32]. This class of drug chemicals present a definite advantage as they only become active in an acidic environment and as a result can selectively target cancer cells in their acidic environment [33]. In addition, they are well tolerated and safe, with severe side effects only rarely reported [34]. They have therefore been successfully used to suppress tumour growth in vitro and in vivo and to overcome drug resistance [24, 33].

**Fig. 2:**
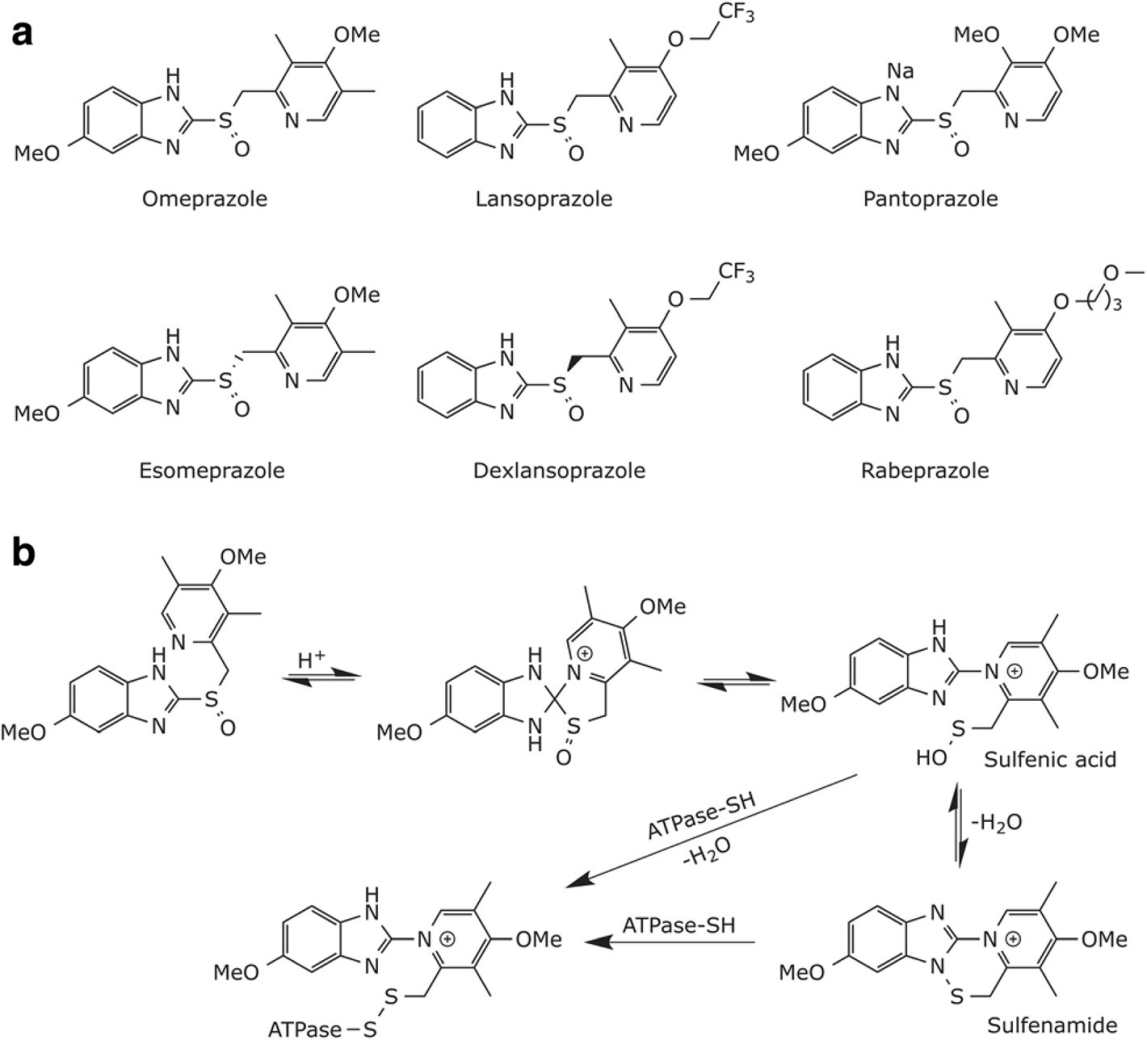
**a** Chemical structure of FDA-approved PPIs. **b** Mechanism of action of PPIs

Proton pump inhibitors (PPIs) are the treatment of choice for acid-related disorders. Omeprazole was the first PPI to be approved by the FDA in 1989 to treat heartburn and other symptoms associated with gastroesophageal reflux disease in humans. FDA approved five more PPIs since then, lansoprazole, pantoprazole, rabeprazole, esomeprazole and dexlansoprazole. The last two drugs are enantiomers of omeprazole and lansoprazole, respectively (Fig. 2a) [35]. PPIs are acid-activated prodrugs [36]: since PPIs are weak bases (pK_a_1 = 3.8-4.9), they are only accumulated in acidic tissues such as parietal cells where the pH can go as low as 1. The concentration of PPIs at the luminal surface of the pump is roughly 1000-fold higher than their concentration in the blood. This selective accumulation of PPIs is largely the reason for their high therapeutic index. Mechanistically, the protonation of the pyridine moiety of PPIs initiates a series of reaction leading to the formation of a sulfenic acid and sulfenamide. The latters are highly reactive thiophilic species which will react covalently with different ATPase cysteines to form stable disulfides, thus inhibiting the acid secretion (Fig. 2b) [37, 38].

### Proton pump inhibitors as cancer treatment

Because cancers develop in an acidic environment and they express high levels of proton pumps, there may be advantages in using PPIs and proton transporter inhibitors (PTIs) as a universal treatment across all forms of cancer with relatively few associated side effects [23]. PPIs have also been linked with an increase in cellular caspase activity and an early accumulation of reactive oxygen species within the cell, all of which result in an increased rate of apoptosis [23]. This direct cytotoxic effect has been supported by several studies showing that PPIs can induce apoptosis in both hematopoietic and solid cancers [5]. Another important effect of PPIs is associated with the modulation of autophagy in cancer cells [39, 40]. Chronic autophagy has been established as a pro-survival adaptation of cells to the acidic tumour microenvironment [41]. Importantly, PPIs have been show to inhibit mTOR signalling, a major regulator of cell growth and autophagy possibly as a consequence of the acidification of the intracellular pH [32].

Beside the direct toxic effects, PPIs may have additional cancer-modulating effects. Increasing the pH of the microenvironment reverses multi drug resistance. There are a number of explanations behind this observation. Firstly, all drugs that are weak base with a pKa around 8 are usually protonated at neutral or low pH. As a result, they cannot cross the bilayer membrane and remain in the acidified milieu (corresponding to the extracellular milieu or intracellular organelles as endosomes or lysosomes). The efflux of protons has been demonstrated to be involved in drug resistance and it is therefore possible to revere drug resistance using either PPIs or PTIs [42]. Secondly, for the anticancer drugs that are not weak bases (but weak acid or neutral) and that therefore should cross the bilayer membrane at low pH, another mechanism involving an increase in the membrane stiffness due to the electrostatic repulsion between negatively charged lipids forming the inner leaflet of the membrane in direct contact with the alkaline cytosol, can block drugs mechanically [17]. In this case, acidifying the cytosol by blocking proton efflux can soften the membrane and reverse the low drug uptake. These pH/membrane dependent mechanisms are well known to work in concert with drug transporters meaning that it is possible to alter drug transporters activity by blocking proton efflux [17].

A study using both human and mouse cell lines found that pre-treatment with PPIs caused the xenograph tumours to reduce in size when compared to using the cytotoxic drug doxorubicin alone which resulted in the tumour remaining static in size [15]. These results suggest that cancer cells were more sensitive to doxorubicin with PPIs pre-treatment.

Increasing the pH of the microenvironment should also lead to a reduction in the amount of proteases being release from cells via exocytosis, thereby reducing the invasiveness and ability of the cancer to form metastasis. A study found that oral supplementation of sodium bicarbonate in xenograft mouse models of metastatic breast and prostate cancers was able to alkalise the acidic microenvironment of cancers resulting in a decrease in the number of metastases [43]. The Whitaker Wellness Institute is currently trialling an ‘alkalinizing therapy’ in their human patients [28, 43].

### Proton pump inhibitors in human and veterinary clinical oncology

PPIs have been met with outstanding success in both in vitro and in vivo pre-clinical studies [23]. Clinical trials have been performed and others are currently underway in human medicine. For instance, studies looked at the use of esomeprazole as chemosensitiser in neo-adjuvant chemotherapy for the treatment of osteosarcoma [15] and as combination treatment with cisplatin and docetaxel in metastatic breast cancer (ClinicalTrials.gov Identifier: NCT01069081). It has been shown that pre-treatment of cancer patients with PPIs prior to the chemotherapy is effective on a number of different tumour types and there is a direct correlation between the ability of the PPIs to change the extracellular pH and the number of H+ transporters in the cell membrane [23].

There is currently limited information as to whether cancers in animals share the same pathological characteristics compared to the human counterpart in terms of tumour microenvironment. There have been a number of studies performed in transgenic mice and cell lines, however very little is known about the pathogenesis of spontaneously occurring tumours in companion animals. There is even less information available in the other species such as horses or exotic animals. Table 1 outlines the species variations in the prevalence of different forms of cancer by site affected. However, many spontaneous cancers in companion animals share the same pathology, molecular characteristics and clinical progression to the human counterparts and therefore it is reasonable to speculate they should respond to PPIs.

**Table 1:** List of most prevalent cancer types in the individual species

Prevalence	Human	Dog	Cat	Horse	Rabbit	Reptile/chelonian	Ferrets
1	Breast/Prostate	Breast	Lymphoid	Skin	Uterine adenocarcinoma	Soft tissue sarcoma	Insulinoma
2	Lung	Skin	Skin	Lymphoma	Lymphosarcoma	Lymphoma	Lymphoma
3	Colon/Rectum	Connective tissue	Breast	Unknown	Embryonal nephroma	Fibrosarcoma	Adrenal

Recently, a clinical trial performed in both dogs and cats by Spugnini et al. [44] supported this hypothesis. The phase I/II study conducted in 34 companion animals has shown that treatment with the PPI lansoprazole combined with chemotherapy resulted in a positive outcome for the majority of the animals, either showing partial or complete response [44].

Therefore, the results from this clinical trial are very positive as the treatment with PPIs resulted in the down staging of tumours with the vast majority of patients having few significant clinical side effects to the high doses of PPIs given, mostly limited to vomiting and diarrhoea secondary to gastric hypochloridia. A case worthy of note within the study is that of a cat with an aggressive lymphoma which had spread to the pancreas and was unresponsive to chemotherapy. The cat went into complete remission after receiving the alkalizing treatment and remained disease free in excess of one year.

More recently, it has been shown that alkalization with a water alkalizer and high dose of lansoprazole increased the efficacy of metronomic chemotherapy in a cohort of dogs and cats with advanced cancer disease [44, 45]. Although this study had some limitations including low number of patients and inability to proper measure the variation of pH in the alkaline cohort, it shows that the manipulation of tumor microenvironment can be exploited in metronomic chemotherapy as well.

The direct cytotoxic effect of the PPIs may present the opportunity for an alternative treatment if owner’s financial constraints cannot allow for expensive chemotherapy treatments or in case of tumour chemoresistance.

**Fig. 3:**
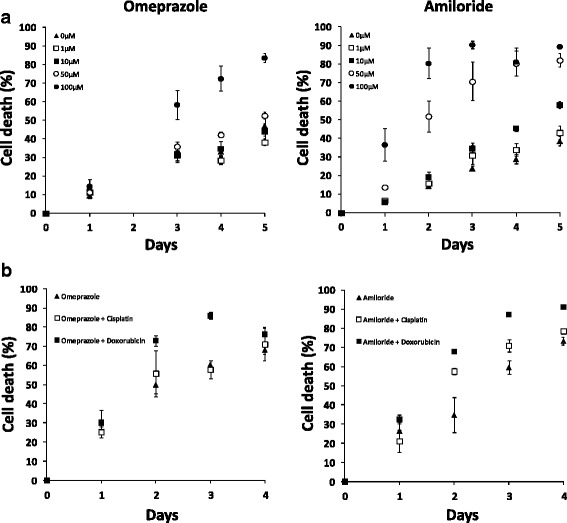
Dose response toxicity curve of canine osteosarcoma D17 sarcospheres treated with omeprazole and amiloride, as assessed by flow cytometry (**a**). Effect of pre-treating sarcospheres with omeprazole and amiloride prior to treatment with doxorubicin and cisplatin versus chemotherapeutic drug alone (**b**)

This is supported by evidence collected during in vitro preliminary studies performed by the authors which showed cellular death was increased in a dose dependent manner using the PPI omeprazole and the NHE blocker amiloride in canine osteosarcoma cells (Fig. 3a). The study performed in chemoresistant sarcospheres derived from canine osteosarcoma cells shows a dose dependent toxic response to the PPI and NHE blocker. In addition , the results show increased cell death when the sarcospheres were pre-treated with PPI/NHE blocker compared to using doxorubicin or cisplatin alone (Fig. 3b).

An added advantage of using PPIs in veterinary medicine is its ease of handling. There are no strict health and safety guidelines set in place as with cytotoxic drugs. Currently, caution must be exerted when preparing and handling chemotherapy drugs, and care must be taken by owners and veterinary staff not to come into contact with the animals bodily fluids for several days after chemotherapy treatment. No specialist facilities are required for PPIs and they could be easily available to all clinicians and owners.

### Two case reports interfering with the Warburg effect in cancer

Some of the authors of this manuscript are currently treating several veterinary patients with a combination of alkalizing therapy and chemotherapy (classic systemic, loco-regional or metronomic). The outcomes of two paradigmatic cases are reported below.

### Case report 1

**Fig. 4:**
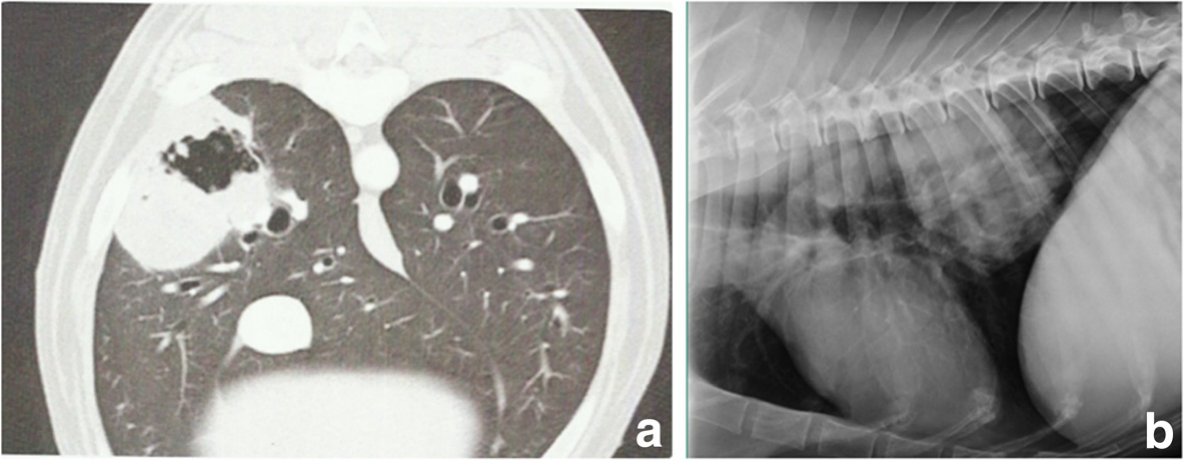
Metronomic chemotherapy with patient alkalization in a 15 year old mixed breed with lung carcinoma. **a** Tumor appearance at presentation (CT scan imaging). **b** Tumor appearance after 14 months of therapy (thoracic radiograph imaging)

A 15 year old male mixed breed dog was referred for oncology consult due to persistent coughing that lasted in excess of one month, unresponsive to symptomatic therapy. The dog was quiet, alert and responsive at physical exam, cardiac function upon auscultation was within normal limits, in the right thoracic side respiratory sounds were muffled and there was a 5 cm area were the sounds could not be appreciated at all. Cough was easily induced upon tracheal stimulation. Haematological analysis and biochemical profile showed a mild anaemia (5.2 x 10^6^/μL RBC with a lower limit of 5.5). Chest radiographs evidenced a mass in the left lung. At this point a CT scan study was deemed necessary to perform a biopsy under guidance (Fig. 4a). The report came back with a diagnosis of bronchogenic carcinoma. The treatment options were discussed with the owner and included lobectomy combined with systemic chemotherapy, systemic chemotherapy alone or metronomic chemotherapy with alkalinisation of the patients. The owner, due to emotional and financial issues chose the third option. The patient was treated with a combination of daily cyclophosphamide, piroxicam and lansoprazole as previously described [45] combined with a water alkalizer. The therapy was well tolerated and the cough subsided, while the patient showed an increased activity level as well as improved quality of life. Side effects were confined to 3 episodes of grade 2 gastrointestinal toxicoses [46] and a mild worsening of the anemia after 8 months of therapy (RBC 4.8 × 10^6^/μL). After 14 months the patient is still in a condition of stable disease and is monitored with chest radiographs every two months (Fig. 4b).

### Case report 2

**Fig. 5:**
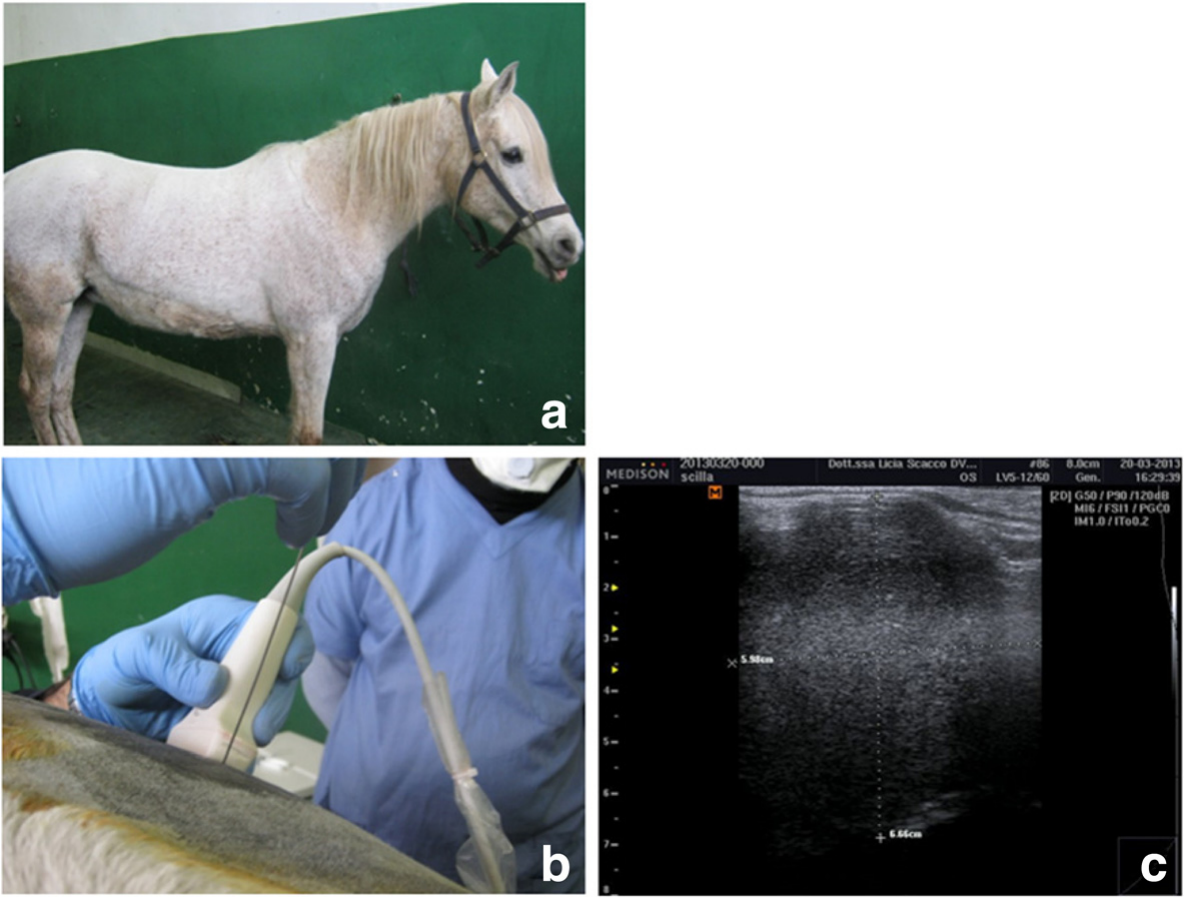
Loco-regional chemotherapy with patient alkalization in a 10 year old Arab mare. **a** Patient at presentation. **b** Ultrasound-guided intralesional chemotherapy with alkalization. **c** Tumor appearance after the 4th session

A 10 year old Arabian mare was referred for multiple muco-cutaneous melanoma and for failure at conceiving. At presentation the horse was bright alert and responsive (Fig. 5a) and presented several melanomas ranging in size from 2 to 8 cm. Ultrasonographic examination was performed showing multiple lymph-nodal metastasis within the abdomen, some of them compressing the uterus and adnexa. Systemic chemotherapy treatment with platinum compounds was deemed unrealistic due to the widespread disease and related expenses with this therapy. At this point, intralesional chemotherapy with CDDP was offered as a palliation. The first course did not result in any measurable response and patient alkalization was then suggested. Considering the amount of PPI or H_2_ blockers needed to achieve this goal, a different approach was proposed: intralesional administration of bicarbonate combined with diet supplementation with the same substance since it is palatable to the horses. In this patient were performed 4 sessions of intralesional 8.4 % (w/v) bicarbonate solution followed by intralesional CDDP 0,5 % (w/v) solution under ultrasonographic guidance (Fig. 5b and c). This therapy yielded a 50 % lymph nodes tumour reduction after 3 sessions. The treatment was discontinued after the 4th session due to financial concerns. The horse has been kept on bicarbonate supplementation as maintenance and is actually experiencing a robust partial remission lasting in excess of two years. Throughout this period the mare has been showing a good quality of life. Side effects secondary to the therapy have not been reported. Alkalization obtained through diet supplementation or through the administration of pump inhibitors can be a valuable strategy to improve the efficacy of standard as well as non-conventional chemotherapy in veterinary oncology.

## Conclusions

The importance of Warburg effect in cancer including the role of proton pumps is now underlined almost every day in the cancer literature [47–54]. PPIs are amongst the most commonly prescribed drugs in human medicine and have gone through the process of rigorous safety testing and monitoring. Very few clinical side effects have been reported at higher doses. In case controlled studies, long term treatment with PPIs was associated with increased risk of bone fracture. Some reports have suggested increased risk of gastric or colon cancer, but these studies are still inconclusive [5]. Therefore it is reasonable to justify the continued investigation into the use of this class of drugs for the treatment of cancer in humans and companion animals. They may provide an alternative or additional source of therapy which could result in more effective and lower cost treatments. They could potentially form part of a universal treatment which may have direct benefits in treating a number of different cancer types while overcoming problems associated with chemotherapy, such as drug resistance and patient discomfort. None of the PPIs is currently licenced for use in veterinary medicine and more research is needed to support their value as new treatment for veterinary cancer patients.
